# Expanded Antigen-Specific Elimination Assay to Measure Human CD8^+^ T Cell Cytolytic Potential

**DOI:** 10.1002/cpz1.1109

**Published:** 2024-07

**Authors:** David R. Collins, Mpho J. Olatotse, Zachary J. Racenet, Umar Arshad, Elif Çakan, Gaurav D. Gaiha, Kiera L. Clayton, Bruce D. Walker

**Affiliations:** 1 Ragon Institute of MGH, MIT & Harvard, Cambridge, MA, USA; 2 Howard Hughes Medical Institute, Chevy Chase, MD, USA; 3 Division of Gastroenterology, Massachusetts General Hospital, Boston, MA, USA; 4 Department of Pathology, University of Massachusetts Chan Medical School, Worcester, MA, USA; 5 Institute for Medical Engineering and Sciences, MIT, Cambridge, MA, USA

**Keywords:** Cytotoxicity, human CD8^+^ T cells, epitope-specific, T-cell memory, flow cytometry

## Abstract

Durable cellular immunity against pathogens is dependent upon a coordinated recall response to antigen by memory CD8^+^ T cells, involving their proliferation and the generation of secondary cytotoxic effector cells. Conventional assays measuring *ex vivo* cytotoxicity fail to capture this secondary cytolytic potential, especially in settings where cells were not recently exposed to their cognate antigen *in vivo*. Here we describe the Expanded Antigen-Specific Elimination Assay (EASEA), a flow cytometric endpoint assay to measure the capacity of human CD8^+^ T cells to expand *in vitro* upon antigen re-exposure and generate secondary effector cells capable of selectively eliminating autologous antigen-pulsed target cells across a range of effector-to-target ratios. Unlike alternative assays, EASEA avoids the hazards of radioactive labeling and viral infection and can be used to study responses to individual or pooled antigens of interest.

Basic Protocol 1: Expanded Antigen-Specific Elimination Assay

## INTRODUCTION:

Adaptive cellular immunity against intracellular pathogens and tumors is mediated by cytotoxic CD8^+^ T cells, whose T cell receptors (TCRs) recognize antigenic peptides presented by class I major histocompatibility complex proteins on the surface of infected cells. Such CD8^+^ T cells exercise effector function by their release of granules containing the cytotoxic effector molecules perforin and granzyme B to cause apoptosis of the target cells [reviewed in (Russell & Ley, 2002)]. Upon antigen clearance, CD8^+^ T cells persist in a functional but quiescent memory state characterized by low expression of cytotoxic effectors and high proliferative potential. These functional cells respond to antigen re-exposure via programmed T cell differentiation pathways that result in the generation of secondary effector cells with high cytolytic capacity (Migueles et al., 2020). However, under some conditions, some CD8^+^ T cells persist in a dysfunctional state characterized by markedly reduced proliferative potential and diminished expression of cytotoxic effector molecules [reviewed in (Wherry, 2011)]. Such dysfunctional quiescent CD8^+^ T cells may be indistinguishable from functional quiescent CD8^+^ T cells by conventional assays usually used to measure *ex vivo* cytotoxicity. This is especially true for cells collected from tissues where expression of cytolytic effector proteins is further suppressed under homeostatic conditions marked by the absence of active pathogen replication and inflammation (Collins et al., 2023; Reuter et al., 2017). However, *in vitro* re-exposure to antigen such as that employed in the assay to be described enables robust assessment of the secondary cytotoxic potential of memory CD8^+^ T cells and can distinguish functional from dysfunctional cells irrespective of recent *in vivo* antigen exposure.

Here we describe the Expanded Antigen-Specific Elimination Assay (EASEA), a flow cytometric method to quantitatively measure the ability of human antigen-specific memory CD8^+^ T cell responses to eliminate autologous peptide-pulsed target cells during a four-hour co-incubation after six days of peptide-specific effector cell expansion. EASEA provides numerous advantages relative to alternative cytotoxicity assays such as chromium release (Brunner, Mauel, Cerottini, & Chapuis, 1968), target cell apoptotic activity (Fischer, Andreesen, & Mackensen, 2002; Jerome, Sloan, & Aubert, 2003; Liu et al., 2002), and virus inhibition (Fauce, Yang, & Effros, 2007; Saez-Cirion, Shin, Versmisse, Barre-Sinoussi, & Pancino, 2010; Yang et al., 1997), avoiding the hazards of radiolabeling and virus infection while measuring the cytolytic potential of antigen-specific memory CD8^+^ T cell responses against specific antigens presented on autologous target cells. In this protocol we describe the assay using HIV epitope-specific CD8^+^ T cell effectors derived from peripheral blood and autologous CD4^+^ T cell targets pulsed with cognate peptide. However, EASEA is adaptable for use with various target cell types pulsed with individual or pooled peptide antigens as well as with primary human cells derived from different tissue origins.

## CAUTIONS:

### CAUTION:

Primary human cells should be obtained only after informed consent under protocols approved by institutional review for human subjects research and should be handled under appropriate Biosafety Level 2 (BSL-2) or higher sterile conditions.

## STRATEGIC PLANNING:

As EASEA is not suitable for high-throughput response screening due to its relatively cell-intensive requirements, individual antigen-specific CD8+ T cell responses of interest should be identified beforehand using screening assays such as the immunospot assay. Alternatively, pooled peptides covering a protein or set of proteins of interest may be used.

## BASIC PROTOCOL 1: Expanded Antigen-Specific Elimination Assay

**Figure 1:**
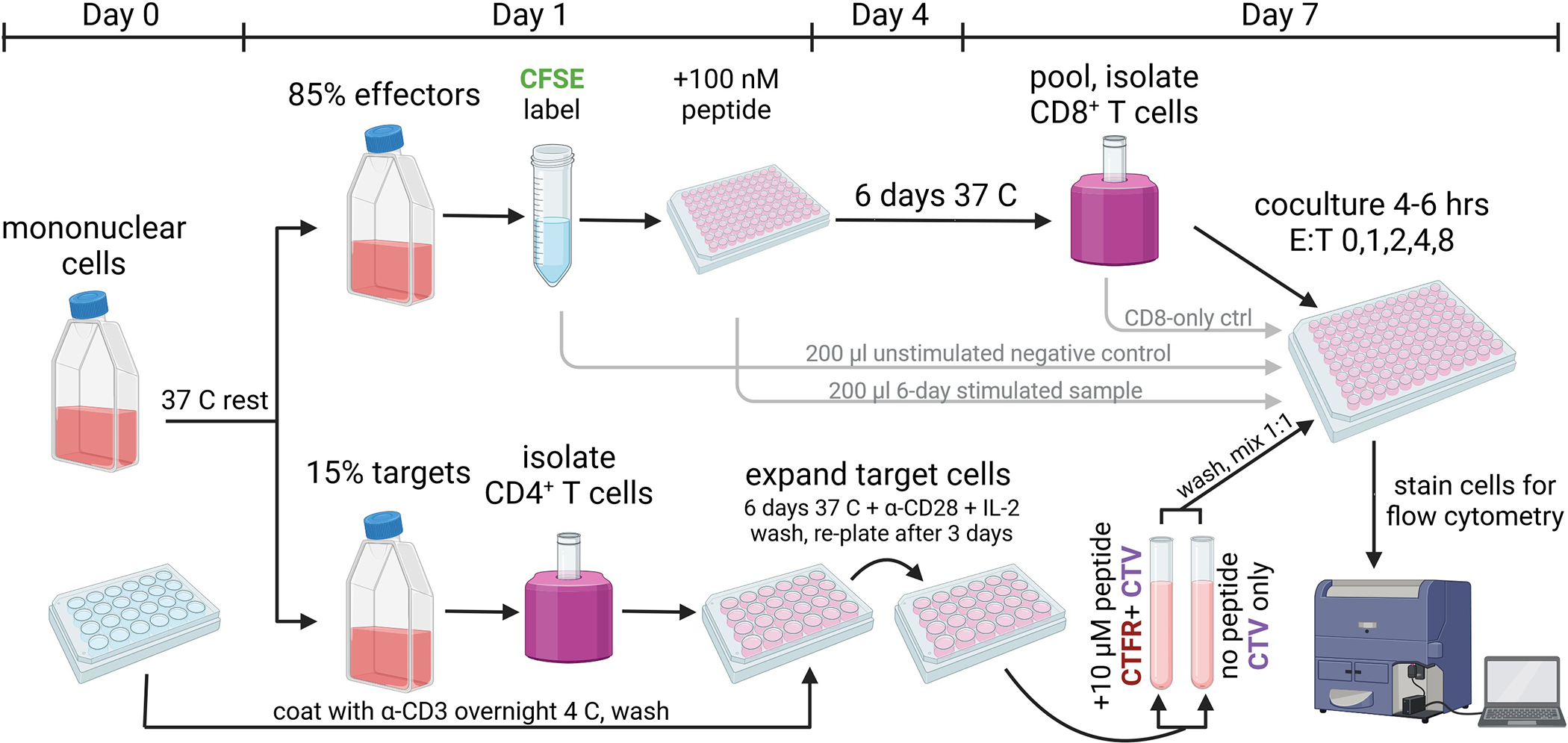
Schematic overview of assay. Overview and timeline of the Expanded Antigen-Specific Elimination Assay protocol. Created using Biorender.com.

This demonstration protocol focuses on the assay of cytotoxic T cells developing in people with HIV infection and thus harboring HIV antigen-specific CD8^+^ T cells. It begins with peripheral blood or tissue-derived mononuclear cells isolated from research participants and then split into two pools for subsequent culture (Fig. 1). The first pool of cells, the effector cell pool, is stimulated with antigenic peptide(s) of interest in the absence of exogenous cytokines; this allows antigen-specific CD8^+^ T cell (effector cell) proliferation that can be subsequently tracked by dilution of carboxyfluorescein (CFSE). The second pool of cells, the target cell pool, is nonspecifically expanded via TCR stimulation after isolation of target cells. 50% of these cells are loaded with the cognate peptide(s) of interest and labeled with a unique fluorescent dye for tracking of their selective elimination across varying effector:target (E:T) ratios during a four-hour coculture. When implemented successfully, the assay measures titratable reductions in residual peptide-pulsed target cells by secondary effector CD8^+^ T cells at increasing E:T ratios, thus enabling quantitation and comparison of antigen-specific cytolytic potential via area-under-curve (AUC) analyses.

### Materials:

Non-treated polystyrene 24-well plates (Corning, Cat# 3738)

Ultra-LEAF purified anti-CD3 antibody (clone OKT3, Biolegend, Cat# 317304)

Carbonate Coating Buffer (Thermo, Cat# CB01100, or alternatively see recipe in Reagents and Solutions)

Plate seals (MP Biomedicals, Cat# 0976401C2)

R10 culture media (see recipe in Reagents and Solutions)

Micrococcal nuclease S7 (Sigma, Cat# 10107921001)

25 cm^2^ cell culture flasks (Corning, Cat# 430639)

EasySep CD4^+^ T cell isolation kit (StemCell Technologies, Cat# 17952)

FACS buffer (see recipe in Reagents and Solutions)

5 ml polystylene tubes (Corning, Cat# 352052)

Sterile reagent reservoirs (Argos Technologies, Cat#B3110–50, or similar)

Ultra-LEAF purified anti-CD28 antibody (clone CD28.2, Biolegend, Cat# 302902)

Recombinant human IL-2 (R&D, Cat# 020-IL-010)

TC-treated polystyrene 24-well plates (Corning, Cat# 3524)

96-well polystyrene round-bottom plates (Corning, Cat# 3879)

Peptide(s) of interest (200 μM working stocks diluted in RPMI, custom-synthesized or commercially purchased)

CellTrace CFSE (Thermo, Cat# C34554)

PBS (Corning, Cat# 21040CM)

CellTrace FarRed (Thermo, Cat# C34564)

CellTrace Violet (Thermo, Cat# C34557)

EasySep CD8^+^ T cell isolation kit (StemCell Technologies, Cat# 17953)

LiveDead Near-IR fixable viability dye (Thermo, Cat# L34975)

BV605-conjugated anti-CD3 (clone SK7, Biolegend, Cat# 344836)

BV711-conjugated anti-CD4 (clone RPA-T4, Biolegend, Cat# 300557)

BUV395-conjugated anti-CD8 (clone RPA-T8, BD Biosciences, Cat# 563795)

Cytofix Fixation Solution (BD Biosciences Cat# 554655, or 4% paraformaldehyde)

Optional: APC-conjugated pHLA multimer(s) of interest (custom-synthesized or commercially purchased)

Biosafety Level 2 cabinet (BL2+ if protocol is adapted for use of target cells infected by HIV-1)

Tabletop centrifuge with conical tube and plate rotors

37 °C, 5% CO_2_ incubator

4 °C refrigerator

EasySep magnets (StemCell Cat# 18000)

200 μl multichannel pipette

Flow cytometer (BD Fortessa or Symphony 5-laser with FACSDiva software, or similar)

FlowJo analysis software (BD Biosciences)

### Day 0 [1–1.5 hours]:

1. Prepare plate for target cell expansion: Coat wells of a *non-treated* 24-well plate with 400 μl/well of a 2 μg/ml solution of anti-CD3 in carbonate coating buffer. Seal the plate and incubate overnight at 4 °C.
  This step will be used for the expansion of CD4^+^ T cell targets. If a different target cell type is desired, an alternative expansion method may be used or expansion may be foregone. If uncultured target cells are desired, a second vial of autologous frozen cells may be thawed prior to coculture.
2. Isolate mononuclear cells from peripheral blood or tissue specimens obtained from participants of interest after informed consent under IRB-approved protocols. If using cryopreserved specimens, thaw in R10 using established cell thawing protocols. We recommend adding thawed cells to 10 ml R10 with 10 U/ml nuclease and centrifugation at 800 RCF for 3 min, then resuspending in R10 at approximately 1–2 million cells/ml in an upright T25 flask for overnight rest at 37 °C 5% CO_2_ to allow for optimal cell recovery. Count viable cells using a microscope and hemacytometer or automated cell counter.
  Alternatively, if using fresh specimens, prepare mononuclear cells using established density gradient centrifugation protocols and proceed to step 3.

### Day 1 [1.5–2.5 hours]:

- Establish separate cell pools (derived from mononuclear cells prepared above) for subsequent effector and target cell cultures: Count cells. For each response of interest, use 85% of cells (or approximately 4–30 million cells) for peptide stimulation of effector cells, and 15% of cells (or approximately 1–6 million cells) for nonspecific target cell expansion.
- Isolate target cells: Isolate CD4^+^ T cells from the designated target cell pool using negative selection kit as per manufacturer’s instructions. Count cells.
- Expand isolated target cells: Remove seal from anti-CD3-coated 24 well plate. Remove anti-CD3 solution by aspiration and gently wash plate twice with 1 ml/well PBS to remove residual carbonate coating buffer.
- Add 400 μl of pre-warmed R10 + 10 ng/ml (~100U/ml) IL-2 to the washed wells.
- Centrifuge isolated CD4^+^ T cells at 800 RCF for 3 min and resuspend at a concentration of up to 2 million cells/ml in R10 + 10 ng/ml IL-2 and add 4 μg/ml of anti-CD28 antibody. Add cells in an additional 400 μl to each well of the 24 well plate (final volume 800 μl). Incubate at 37 °C 5% CO_2_ for 3 days.
- Label effector cells in the designated effector cell pool: First, prepare 5X CFSE solution by thawing one vial of lyophilized CFSE, resuspending the contents in 18 μl DMSO, vortexing the mixture and adding to 20 ml PBS. Second, centrifuge cells in the effector cell pool at 800 RCF for 3 min in a conical tube and resuspend in 800 μl PBS. Add 200 μl 5X CFSE solution, mix, and incubate at 37 °C 5% CO_2_ for 20 minutes. After incubation, quench any remaining dye in solution by adding 10 ml pre-warmed R10.
- Expand antigen-specific effector cells: Centrifuge CFSE-stained mononuclear cells in the effector cell pool at 800 RCF for 3 min then resuspend in R10 at 1 million cells/ml. Split off 200 μl into one well of a 96-well round-bottom plate as an unstimulated negative control. Add a final concentration of 100 nM HLA-optimal peptide(s) of interest to expand antigen-specific CD8^+^ T cells in the remaining mononuclear effector cell pool, then plate 200 μl/well (200,000 cells/well) of the cell suspension into the 96-well plate(s). Incubate at 37 °C 5% CO_2_ for 6 days.
  If multiple responses are to be tested separately, split cells into multiple cultures, one for each peptide. If testing a polyclonal response to multiple peptides, add multiple peptides to same culture at 100 nM each.

### Day 3 [0.5–1 hour]:

- Expand target cells: After 3 days of stimulation, remove CD4^+^ T cells from coated wells, count cells, centrifuge at 800 RCF for 3 min, and resuspend cells in pre-warmed R10 + 10 ng/ml IL-2 at approximately 1 million cells/ml. Plate 1 ml/well in a new TC-treated 24-well plate. Incubate at 37 °C 5% CO_2_ for 3 days.

### Day 6 [6–8 hours (hands-on: 2–4 hours)]:

- Peptide-pulse target cells: After 3 additional days of expansion post-stimulation of CD4^+^ T cell targets, resuspend, pool and count cells. Split cells into two conical tubes, leave one tube un-pulsed with peptide and add 10 μM peptide(s) of interest to the second tube. Incubate at 37 °C for 25 min.
  Note: If testing multiple responses separately, split peptide-loaded cells into multiple cultures, one for each peptide. If testing a polyclonal response to multiple peptides, add multiple peptides to same culture at 10 μM each.
- Isolate effector cells: After 6 days of antigen-specific expansion, pool all wells of mononuclear cell populations containing CD8^+^ T cells expanded by each peptide by resuspending well contents using a multichannel pipettor and transferring into reagent reservoirs, then into a conical tube. After pooling, save 200 μl of unstimulated and peptide-stimulated mononuclear cells in one well of a 96-well round-bottom plate at 37 °C to later assess antigen-specific expansion. While target cells are incubating with peptide(s), count and isolate CD8^+^ T cells from the remaining CFSE-stained, peptide-expanded mononuclear cell populations using a magnetic negative selection kit as per the manufacturer’s protocol. Count isolated CD8^+^ T cells.
  Note: If testing multiple responses separately, perform isolations for each peptide-specific culture separately.
- Label target cells: Prepare stocks of CellTrace Far Red (CTFR) and CellTrace Violet (CTV) dyes by resuspending in 50 μl DMSO per vial. After 25 min of incubation with/without peptide(s) as in Step 11, add 1 μl CTV per ml to both unpulsed and peptide-pulsed cells in 1 ml R10, and add 1 μl CTFR per ml to *only the peptide-pulsed* cells in 1 ml pre-warmed R10. Incubate at 37 °C 5% CO_2_ for 5 min, then wash twice with 10 ml R10. Resuspend final pellets in 1 ml R10 each and combine pulsed and unpulsed cells at a 1:1 ratio.
- Coculture effector and target cells: Prepare a 96-well round-bottom plate for coculture across varying effector:target ratios. A range of 25,000 to 150,000 target cells/well is recommended, depending upon effector and target cell yields. Assuming sufficient cells for 50,000 target cells/well, dilute mixed target cells to a concentration of 500,000 cells/ml in R10 and plate 100 μl per well; centrifuge and resuspend effector cells at 4,000,000 cells/ml in R10 and plate 12.5 μl for E:T 1:1, 25 μl for E:T 2:1, 50 μl for E:T 4:1, and 100 μl for E:T 8:1. Add another 12.5 μl to an additional well as an effector-only control. Add R10 to bring all wells to 200 μl final volume and incubate at 37 °C 5% CO_2_ for 4–6 hours to allow for cytotoxic elimination of target cells by effector cells.
  To minimize pipetting error, an alternative approach is to prepare serial two-fold dilutions of effector cells in a separate 96-well plate and transfer into target cells using a multichannel pipette.
- Flow cytometric staining: After coculture, centrifuge plate at 800 RCF for 3 min. Flick plate (or multichannel-aspirate, if preferred) to discard supernatant into the appropriate biohazard waste container inside the biosafety cabinet. Resuspend cells in FACS buffer. Optionally, before surface staining, add 2–4 nM APC-conjugated peptide-HLA (pHLA) multimer(s) of interest to the effector-only and/or CFSE proliferation samples for 15 min at 4 °C to assess antigen-specific TCR surface expression and frequency of antigen-specific effector cells. Add cocktail of BV605-conjugated anti-CD3, BV711-conjugated anti-CD4, BUV395-conjugated anti-CD8 (each at 1:100 final dilution) and Live-Dead Near-IR (resuspended in 50 μl DMSO – unused portion can be refrozen at −20C for future use) at 1:1000 final dilution. Incubate for 30 minutes at 4 °C then wash with 200 μl/well FACS buffer.
  If desired, unpulsed effector cells may be left unstained as a single-stained CFSE compensation control. Do not add multimer to samples with labeled target cells as APC fluoresces in the same channel as CTFR and cell-cell interactions will likely be blocked by TCR-pHLA multimer binding.
- Compensate fluorescence overlap and acquire data using a flow cytometer, then analyze data using flow cytometry analysis software as discussed below in the Understanding Results section.
  Note: Flow cytometry data can be acquired the same or next day if stored at 4 °C protected from light.

## REAGENTS AND SOLUTIONS:

### Carbonate Coating Buffer

8.4 g NaHCO_3_ (Sigma, Cat# S6014)

3.56 g Na_2_CO_3_ (Sigma, Cat# 223530)

1 L ddH_2_O

Adjust pH to 9.5, filter 0.2 μm

Store ≤1 year at 4 °C

### FACS buffer

500 ml PBS (Corning, Cat# 21040CM)

10 ml (2%) FBS (Sigma, Cat# F4135)

Store ≤1 month at 4 °C

### R10 culture media

500 ml RPMI (Sigma, Cat# R0883)

50 ml (10%) FBS (Sigma, Cat# F4135)

5 ml penicillin/streptomycin (Corning, Cat# MT30002CI)

5 ml (2 mM) L-glutamine (Corning, Cat# MT25005CI)

5 ml (10 mM) HEPES (Corning, Cat# MT25060CI)

Store ≤1 month at 4 °C

## COMMENTARY:

### Critical Parameters:

The controls recommended in this protocol are critical for interpretation of results. Pre-mixing separately labeled peptide-pulsed and unpulsed cells and comparing increasing E:T ratios against an E:T of 0:1 as a control allows for measurement of titratable peptide-specific killing without confounding effects of nonspecific cell death or errors in pipetting or cell counting.

We have optimized the protocol for use with 25,000 to 150,000 target cells per well, but for limiting samples we have attempted using as low as 10,000 target cells per well with success. Because peptide stimulation downregulates TCR from the surface of effector cells, the incubation time after stimulation has been optimized. We and others have found that TCR re-expression as well as perforin and granzyme B expression are maximal at day 6 post-stimulation (Migueles et al., 2020), allowing for the assessment of maximal secondary cytotoxicity. We recommend the inclusion of pHLA multimer staining in a control well to distinguish between biological (e.g., lack of responsiveness to stimulation) and technical (e.g., responsiveness but suboptimal TCR re-expression) reasons for a negative result. Inclusion of this control also allows for the option of normalizing secondary cytotoxicity for the size of the pre- or post-expansion antigen-specific CD8^+^ T cell pool to calculate the antigen-specific E:T ratio and measure per-cell in addition to endpoint cytotoxicity, which is influenced by both the size and functional capacity of the response.

EASEA was optimized to measure responses against individual HLA-optimal peptides of 8–12 amino acids in length; however, it may also be used with pooled antigenic peptides. For longer peptides that require endogenous processing and presentation, longer incubation times with target cells may be required prior to coculture. In our experience, a concentration of 100 nM peptide yields superior responsiveness and TCR re-upregulation by day 6 relative to higher or lower concentrations. At this concentration, a peptide washout step was not necessary to achieve maximal TCR re-expression. Our protocol recommends overloading target cells with 10 μM of peptide during pulsing, but this concentration may be reduced and/or replaced by strategies to endogenously express cognate antigen via infection or transfection. In addition, the pulsing concentration may be titrated experimentally to measure the antigen-sensitivity of secondary cytotoxic CD8^+^ T cell responses, which recent evidence suggests may be particularly important for evaluation of vaccine-induced responses (Migueles et al., 2023). While we have optimized the assay using autologous target cells, it is also possible to use target cell lines provided they express the relevant HLA(s) for killing by the CD8^+^ T cell response(s) of interest; this may be useful if it is desired to control for inter-subject heterogeneity in target cell properties that may influence susceptibility to cytolysis. If the primary target cells used may contain replication-competent virus expressing the peptide(s) of interest, antivirals may be included in the target cell culture to prevent viral spread among target cells during expansion, which may otherwise reduce assay sensitivity by enabling antigen presentation in unpulsed target cells.

Like most functional assays involving *in vitro* cell culture, we have observed that lot-specific variations in serum added to culture media can impact results. Therefore, we suggest only comparing results obtained using the same serum lot and/or pre-screening serum lots for comparable performance when switching to a new lot. In our experience, it is sufficient to pre-screen new serum lots using the CFSE dilution and viability readouts on whole PBMCs. The suggested flow cytometry panel can be adjusted to replace fluorophores based on available equipment and/or cost limitations. The assay may also be modified to assess immediate ex vivo cytotoxicity, including by NK cells (Clayton et al., 2021), and elimination of virus-infected target cells and/or non-CD4^+^ T cell targets, as demonstrated for CD8^+^ T cell elimination of HIV-infected macrophages (Clayton et al., 2018).

### Troubleshooting Table:

Advice for troubleshooting problems that could potentially arise during the assay are summarized in Table 1.

### Understanding Results:

Memory CD8^+^ T cell responses with robust secondary cytotoxic potential will display greater specific elimination of autologous target cells displaying their cognate peptide(s). Conversely, T cell responses with poor cytotoxic potential yield little to no specific elimination. For example, in the context of HIV infection, spontaneous control of viremia is strongly associated with the proliferative and cytolytic potential of antigen-specific CD8^+^ T cell responses against nonmutated epitopes [reviewed in (Collins, Gaiha, & Walker, 2020)]. EASEA provides data on proliferation via CFSE dilution and the subsequent specific elimination of autologous peptide-pulsed target cells. We recently used EASEA to reveal a longitudinal decline in cytotoxic potential of antigen-specific CD8^+^ T cells preceding aborted spontaneous control of HIV viremia using samples from peripheral blood and lymph node excisional biopsies (Collins et al., 2021).

**Figure 2:**
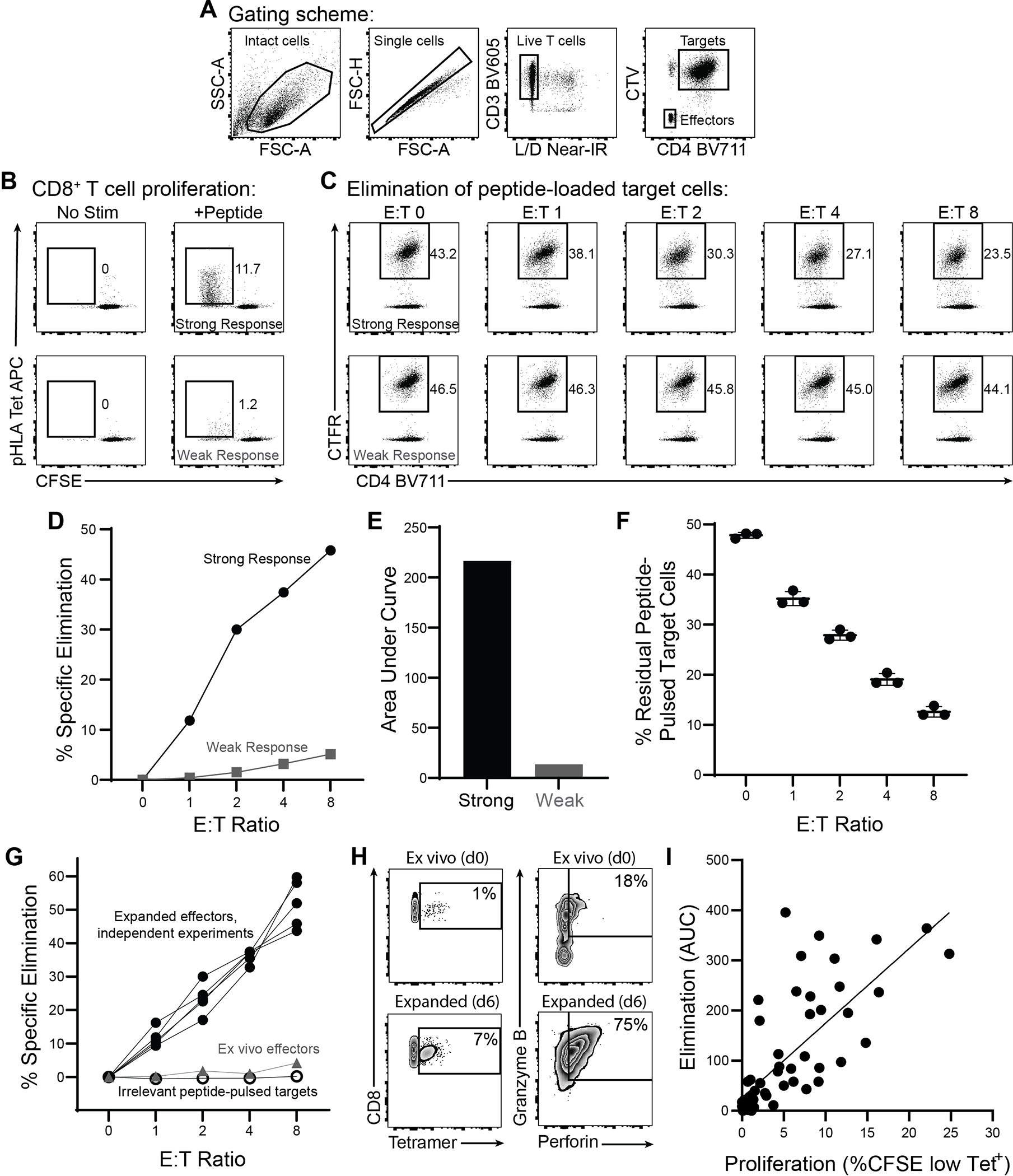
Example results and analyses. **(A)** Suggested gating scheme for data analysis. **(B)** Example proliferation and pHLA multimer staining results for strong and weak responses. **(C)** Example elimination results for strong and weak responses. **(D)** Example plot of percent specific elimination across varying effector:target (E:T) ratios. **(E)** Example comparison of area under the curve (AUC) for strong and weak response elimination data shown in C-D. **(F)** Frequencies of residual peptide-pulsed target cells at the indicated E:T ratios for triplicate wells within the same experiment. **(G)** Percent specific elimination of irrelevant (open symbols) or cognate (closed symbols) peptide-pulsed target cells by peptide-expanded effector cells from five independent experiments (filled circles) or by unexpanded ex vivo effector cells (gray triangles) at the indicated E:T ratios. **(H)** Surface CD8 and pHLA multimer (left) and intracellular perforin and granzyme B (right) staining of effector cells before (ex vivo, day 0) and after (expanded, day 6) peptide expansion. **(I)** Spearman correlation of antigen-specific proliferation (as measured in B) and elimination (as measured in E) among *n* = 57 CD8^+^ T cell responses to HIV peptides.

Here we provide example data for antigen-specific responses with different cytotoxic potential. A suggested analytical gating scheme is provided (Fig. 2A). The extent of proliferation is measured by CFSE dilution relative to an unstimulated negative control, and surface TCR-pHLA binding can be optionally assessed using pHLA multimer staining (Fig. 2B). Specific elimination of peptide-loaded target cells is measured by residual frequency of dye-labeled target cells compared to a negative control in which effector cells were not co-cultured (Fig. 2C). Percent of eliminated target cells (Fig. 2D) at each E:T ratio can be calculated as 100*(1–(residual pulsed target frequency at E:T 1, 2, 4, or 8/ pulsed target frequency at E:T 0)). Elimination across a range of E:T ratios can be quantified by area-under-curve (AUC) analysis (Fig. 2E) to enable summary comparisons between responses.

Results are highly reproducible across replicate wells (Fig. 2F) and independent replicate experiments (Fig. 2G). Specificity is internally controlled by measuring the selective elimination of peptide-pulsed relative to unpulsed cells. As an additional control, target cells pulsed with an irrelevant peptide were not eliminated by cognate peptide-expanded cells, further demonstrating specificity (Fig. 2G). Cognate peptide-pulsed cells were poorly eliminated by unexpanded *ex vivo* effector cells from the same donor, demonstrating the importance of peptide restimulation for assessment of secondary cytolytic potential (Fig. 2G). This was attributable to increases in both the number of and expression of cytolytic effectors perforin and granzyme B among antigen-specific effector cells (Fig. 2H).

EASEA is designed to capture the combined ability of antigen-specific CD8^+^ T cells to proliferate and eliminate target cells, functions that are mechanistically related (Migueles et al., 2002). Consistent with this notion, we observed a strong correlation between CD8^+^ T cell proliferation and specific elimination of autologous peptide-loaded target cells (Fig. 2I). Notably, this correlation is not absolute, including responses with relatively strong elimination despite fewer expanded antigen-specific effectors as well as weaker elimination despite more numerous expanded antigen-specific effectors, suggesting that additional factors beyond the number and expansion capacity of antigen-specific effector cells influence relative cytotoxicity, consistent with recent literature (Rudloff et al., 2023). Because E:T ratios are calculated using total T cells, they importantly do not represent the antigen-specific E:T ratios. Although not required for endpoint measurement of secondary cytotoxic potential, elimination results may be further normalized for relative quantities of antigen-specific effector cells before or after expansion in order to assess relative differences in per-cell elimination capacity.

### Time Considerations:

The assay spans six days and includes approximately ten hours of hands-on time, as indicated in the protocol.

## Figures and Tables

**Table 1.** Troubleshooting Guide for Expanded Antigen-Specific Elimination Assay

Problem	Possible Cause	Solution
No/poor killing	Response is poorly functional	Can be confirmed using CFSE dilution, pHLA multimer surface staining, perforin and granzyme B intracellular staining on day 6
	Suboptimal TCR re-expression after proliferation	Wash out peptide from effector cells after day 3 to prevent restimulation, modify peptide concentration, and/or modify length of incubation
	Insufficient antigen-specific effectors	Increase effector cell number per well and/or decrease target cell number per well

## Data Availability

The data that support this protocol are available in this article.
